# Interplay between porin deficiency, fitness, and virulence in carbapenem-non-susceptible *Pseudomonas aeruginosa* and *Enterobacteriaceae*

**DOI:** 10.1371/journal.ppat.1012902

**Published:** 2025-02-07

**Authors:** Hedi Mammeri, Youssouf Sereme, Eya Toumi, Hélène Faury, David Skurnik

**Affiliations:** 1 Service de Bactériologie, Assistance Publique Hôpitaux de Paris, Hôpitaux Universitaires Paris Centre, Site Cochin, Paris, France; 2 INSERM U1151, CNRS UMR8253, Institut Necker Enfants Malades, Université Paris Cité, Paris, France; 3 Laboratoire de Microbiologie Clinique, AP-HP Centre, Hôpital Necker Enfants Malades, Paris, France; University of Pittsburgh, UNITED STATES OF AMERICA

## Abstract

The increasing resistance of Gram-negative bacteria to last resort antibiotics, such as carbapenems, is particularly of concern as it is a significant cause of global health threat. In this context, there is an urgent need for better understanding underlying mechanisms leading to antimicrobial resistance in order to limit its diffusion and develop new therapeutic strategies. In this review, we focus on the specific role of porins in carbapenem-resistance in *Enterobacteriaceae* and *Pseudomonas aeruginosa*, which are major human pathogens. Porins are outer membrane proteins, which play a key role in the bacterial permeability to allow nutrients to enter and toxic waste to leave. However, these channels are also “Achilles’ heel” of bacteria as antibiotics can also pass through them to reach their target and kill the bacteria. After describing normal structures and pathways regulating the expression of porins, we discuss strategies implemented by bacteria to limit the access of carbapenems to their cytoplasmic target. We further examine the real impact of changes in porins on carbapenems susceptibility. Finally, we decipher what is the effect of such changes on bacterial fitness and virulence. Our goal is to integrate all these findings to give a global overview of how bacteria modify their porins to face antibiotic selective pressure trying to not induce fitness cost.

## Introduction

Outer membrane in Gram-negative bacteria constitutes a major protective barrier against aggressive agents. However, this sturdy barrier must allow nutrients essential for the bacteria to pass through. Porins are key proteins for transporting molecules including nutrients (e.g., sugars, ions and amino acid) through the outer membrane [1]. These outer membrane proteins (OMPs) have a β-barrel structure and form a water-filled channel anchored into the outer membrane [2]. There are three groups of transport-specialized porins: (i) general (or classic or non-specific) porins that allow the passive diffusion of hydrophilic substrates with respect of charge and size (often <600 Da) [2], (ii) substrate-specific porins involved in the passive transport of certain specific solutes [3], and (iii) active transporters such as TonB-dependent receptors involved in the uptake of large substrates (e.g., iron siderophores) [4]. Porins may also be involved in physiological processes other than transport. For example, some are potentially involved in interactions between the peptidoglycan layer and the outer membrane [5,6], or have roles in virulence (by contributing to bacterial adhesion and invasion [7–9] or participating in the bacterial defense by neutralizing host defense mechanisms [10,11]).

However, by forming holes in the outer membrane, porins weaken this physical barrier against exogenous toxic compounds. Indeed, they also allow the entry of many antibiotics into the bacterium. For instance, the β-lactams used in the clinic cross the outer membrane by passing through porins to reach their periplasmic targets, the penicillin-binding proteins (PBPs). The speed with which the required antibiotic concentration can be reached in the periplasmic space is an important effectiveness variable for these antibiotics; it depends mainly on the number and structural integrity of OMPs, but also on the size, charge, and hydrophobicity of β-lactams [12,13].

**Fig 1:**
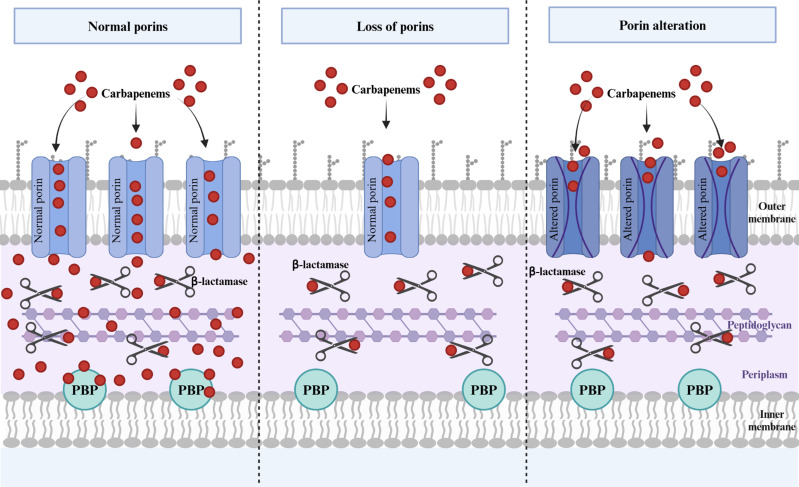
A schematic representation of the combined effect of porin deficiency and β-lactamase production. Left panel. In the wild type, the effective passage through porins leads to a high periplasmic concentration of carbapenems (red spots). The weak carbapenemase activity of the bacterium’s β-lactamases (*i.e.,* ESBLs or AmpC enzymes), symbolized here by scissors, is outweighed by the high antibiotic concentration; the carbapenems bind and inhibit the target penicillin-binding proteins (PBPs). Middle and right panels. In mutant strains, defects in porins (loss of porins or porin alteration) slow the passage of carbapenems into the periplasmic space. The resulting low amounts of carbapenem are hydrolyzed by the bacterium’s β-lactamases, and thus do not reach the target PBPs thus contributing to carbapenem resistance. Created with BioRender.com.

In return, bacteria developed strategies consisting of quantitative or qualitative changes in porins (caused by mutations in regulatory and/or structural genes), which reduce antibiotic entry, and therefore may lead to antibiotic resistance. These changes in permeability are of particular concern because they partly enable the emerging “superbugs” to resist antibiotics. Thus, porins contribute to the multidrug resistance problem – especially when they are responsible for resistance to last-line antibiotics such as carbapenems. The carbapenems constitute a group of β-lactam antibiotics indicated for the treatment of infections caused by multidrug-resistant, Gram-negative bacilli, including *Enterobacteriaceae* and *Pseudomonas aeruginosa*. Carbapenem resistance conferred by changes in porins is usually amplified by the hydrolysis of carbapenems by periplasmic β-lactamases (Fig 1). Indeed, by reducing the carbapenem concentration in the periplasmic space, porin deficiency renders the antibiotics more vulnerable to the weak carbapenemase activity displayed by some β-lactamases present in the periplasmic space, such as extended-spectrum β-lactamases (ESBLs) and AmpC enzymes [14–16]. Although carbapenems have been effective against a large number of clinical isolates for decades, resistance is becoming increasingly common. In 2024, the World Health Organization updated its Bacterial Priority Pathogen List [17], and listed carbapenem-resistant *Enterobacteriaceae* and carbapenem-resistant *P. aeruginosa*, as of “critical” and “high” priority pathogens, respectively, due to their global public health threat.

In addition, because porins are also essential for the entry of nutrients, changes in porins may have an impact on bacterial fitness. There is no consensus on whether carbapenem-resistant isolates are more virulent or less virulent than carbapenem-susceptible isolates. It was long thought that antibiotic resistance had a fitness cost. However, studies such as the comprehensive analysis of saturated bank of mutants of *P. aeruginosa* may have started to challenge this dogma suggesting that porin-deficient strains of *P. aeruginosa*, and *Acinetobacter baumannii* may also be more virulent [18].

In the present review, we sought to reconcile the different points of view and observations by showing that the evolutionary constraints imposed by changes in the porin pattern lead to trade-offs between fitness/virulence and carbapenem resistance in Gram-negative bacilli. To that end, we evaluated the impact of changes in the porin profile on carbapenem resistance, bacterial fitness, and virulence. We focus on two main clinically relevant Gram-negative bacilli: the *Enterobacteriaceae* and *P. aeruginosa*. Furthermore, we looked at how various forms of porin deficiency modulate these bacteria’s pathogenicity and eventually lead to the worst-case scenario: antibiotic-resistant bacteria that are fitter and/or more virulent.

## Mechanisms leading to porin loss or alterations

### Normal structure and regulation of porins involved in carbapenem entry in *Enterobacteriaceae*

**Fig 2:**
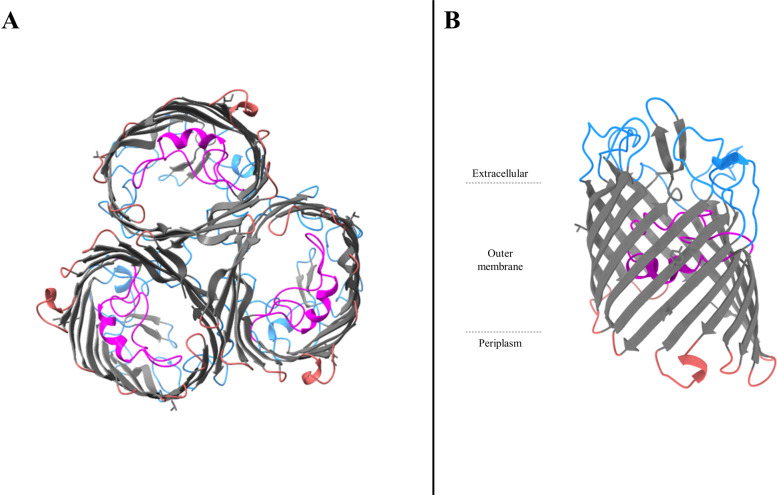
Overall structure of the OmpF porin of *Escherichia coli.* **A**. Cartoons of *E. coli* OmpF homotrimers viewed from the periplasmic space. **B**. Side view of *E. coli* OmpF monomer. Gray, β-strands; blue, extracellular loops and α-helice; magenta, pore-constricting loop L3 and α-helice; red, short periplasmic turns and periplasmic α-helice. These graphics are based on PDB file 2OMF and were drawn by using the program UCSF ChimeraX 1.8 (https://www.rbvi.ucsf.edu/chimerax) [22].

**Fig 3:**
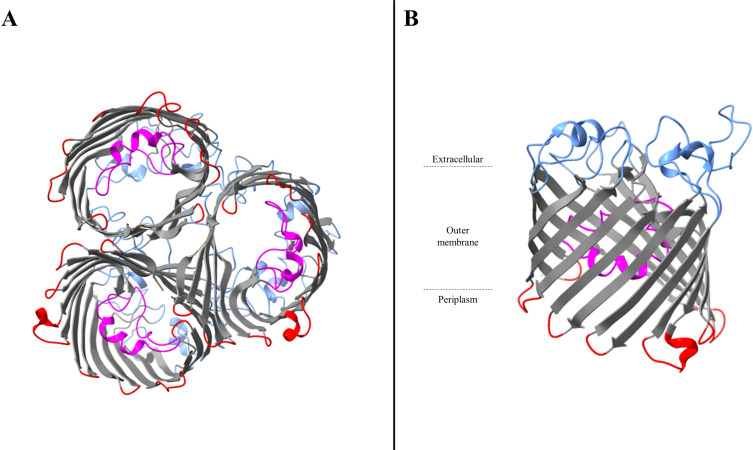
Overall structure of the OmpC porin of *Escherichia coli.* **A**. Cartoons of *E. coli* OmpC homotrimers viewed from the periplasmic space. **B**. Side view of *E. coli* OmpF monomer. Gray, β-strands; blue, extracellular loops and α-helices; magenta, pore-constricting loop L3 and α-helice; red, short periplasmic turns and periplasmic α-helice. These graphics are based on PDB file 2J1N and were drawn by using the program UCSF ChimeraX 1.8 (https://www.rbvi.ucsf.edu/chimerax) [22].

Most of the porins involved in carbapenem entry in *Enterobacteriaceae* are general porins. General porins are major porins in *Enterobacteriaceae*. They are usually composed of homotrimers (Figs 2 and 3). Each monomer has typically a β-barrel structure comprising 16 β-strands linked by extracellular loops and periplasmic turns [2,4,19] (Figs 2 and 3). The third loop (L3) is an essential element. This loop is not exposed at the cell surface, but folds back into the channel and forms a constriction zone through the channel giving it an hourglass shape (Figs 2B and 3B) [2]. This constriction (also known as an eyelet) defines the size of the molecules that can pass through the channel [2]. In addition, this constriction region also conditions the charge of solutes that can pass through due to a strong electric field generating by the folding of L3, with negatively charged (or acidic) residues in L3 facing positively charged (or basic) residues on the opposite barrel wall [20]. Interestingly, this region is highly conserved in *Enterobacteriaceae* [21].

*Escherichia coli* OmpC and OmpF, encoded by *ompC* and *ompF*, respectively, are two major archetypal general porins in *Enterobacteriaceae* [2,23]. The permeability of these porins is different. Indeed, OmpC is less permeable than OmpF. This observation was first attributed to the size of the OmpC pore, which is slightly narrower than that of OmpF [2]. Nevertheless, the presence of more negative charges in OmpC appears to be the main reason for the difference in selectivity between the two porins [23]. Indeed, 10 residues differ in charge in the pore lining region of OmpF and OmpC, with 2 negatively charged residues per monomer in OmpF versus 4 in OmpC [24]. Kojima and Nikaido exchanged these 10 residues that differ between OmpF and OmpC and showed that this exchange swaps the antibiotic permeation properties with OmpC becoming OmpF-like and reciprocally [24]. Thus, because OmpC is more cation-selective than OmpF, this can explain the low permeability of OmpC to anionic β-lactams such as moxalactam, aztreonam, ceftriaxone [25,26]. These proteins’ structure–activity relationships have been extrapolated to homologous general porins produced by other *Enterobacteriacae* species, such as OmpK36 or Omp36 (OmpC-like porin) and OmpK35 or Omp35 (OmpF-like porin) in *Klebsiella pneumoniae* and *Klebsiella aerogenes*, respectively [27–30].

**Fig 4:**
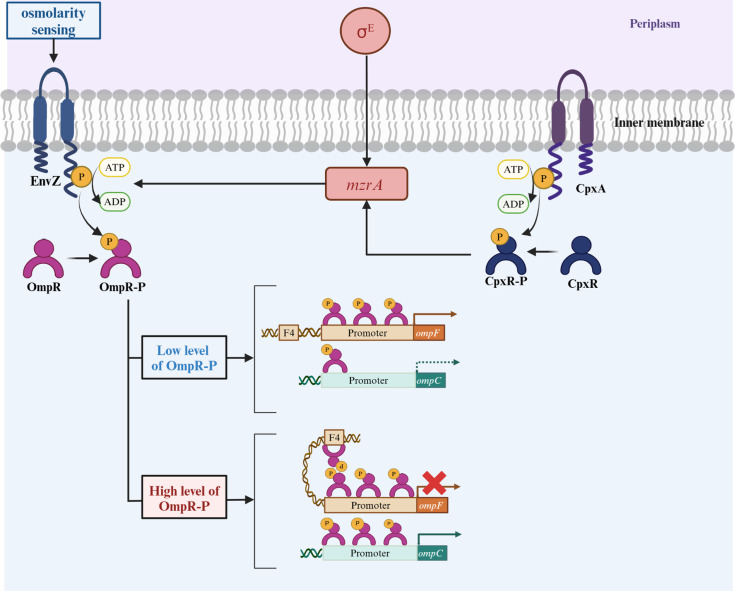
Transcriptional regulation of genes encoding the two porins OmpF and OmpC in *Escherichia coli.* Transcriptional regulation of *ompC* and *ompF* expression by the two component systems EnvZ/OmpR and CpxRA. The preferential expression of one of the two porins depends on the level of OmpR-P which depends on the level of phosphorylation of EnvZ. Under low osmolarity, a low level of OmpR is phosphorylated. It is sufficient to bind to the *ompF* promoter, prompting high levels of OmpF on the membrane, but insufficient to bind to a low-affinity binding sites of *ompC* promoter. In contrast, under high osmolarity more OmpR-P is formed, which eventually binds to all sites of the *ompF* promoter, including F4, creating a loop which represses *ompF* transcription. Meanwhile, OmpR-P also binds with all sites of the *ompC* promoter thus increasing the level of OmpC. Created with BioRender.com.

The enterobacterial general porins’ expression is complex and regulated at the transcriptional and post-transcriptional levels (Fig 4). In *E. coli*, the expression of the two general porins OmpF/OmpC is tightly regulated by the EnvZ/OmpR two-component system (TCS) [31,32]. The level of phosphorylation of the transmembrane sensor kinase EnvZ depends on the environment’s osmolarity [33,34]. Under low-osmolarity conditions, a small amount of EnvZ is phosphorylated, and so a low level of the response regulator OmpR is activated after phosphorylation by EnvZ (Fig 4). A low level of OmpR-P is sufficient to bind to the high-affinity *ompF* promotor site, but insufficient to bind to a low-affinity site like the *ompC* promoter. Consequently, *ompC* is transcribed less than *ompF* under low-osmolarity conditions. Under high-osmolarity conditions, EnvZ actively auto-phosphorylates and has a higher kinase activity. Thus, the amount of OmpR-P is higher; OmpR-P can therefore bind to low-affinity sites and activate *ompC* transcription, which represses *ompF* expression [34] (Fig 4). In high-osmolarity media, *ompC* is transcribed more than *ompF* [33]. Interestingly, this reciprocal regulation might be beneficial in the bacterium’s natural environments. Indeed, in high-osmolarity environments (such as the intestine, where the concentrations of toxic molecules (such as bile salts) are relatively high), OmpC—which has a smaller pore than OmpF—is more expressed and slows down the diffusion of small molecules. Hence, OmpC filters more stringently and can exclude toxic compounds. Conversely, in low-nutrient conditions, OmpF is the major porin for nutrient entry into the bacterium [35]. The amino acid sequences of the OmpR/EnvZ TCS is highly conserved in *Enterobacteriaceae* [36]. Similarly, in *K. pneumoniae*, Fajardo-Lubián and colleagues showed that when the strain grows in low-nutrient, low-osmolarity conditions, OmpK36 is weakly expressed and OmpK35 is strongly expressed [37]. These findings suggest that EnvZ/OmpR TCS could also be involved in the regulation of OmpK36 and OmpK35 expression in *K. pneumoniae*.

In addition, a second TCS (CpxRA, reportedly a major pathway for the outer membrane stress response) regulates general porin expression in *Enterobacteriaceae* [38]. Interestingly, this TCS is connected to EnvZ/OmpR via a small membrane protein MzrA [39,40] (Fig 4). The transcription of *mzrA* is directly activated by the σ^E^ regulon or indirectly by the activated CpxRA system. In turn, MzrA modulates EnvZ/OmpR activity and induces the expression of more OmpR-P. The resulting high level of OmpR-P upregulates *ompC* expression and downregulates *ompF* expression [39].

**Fig 5:**
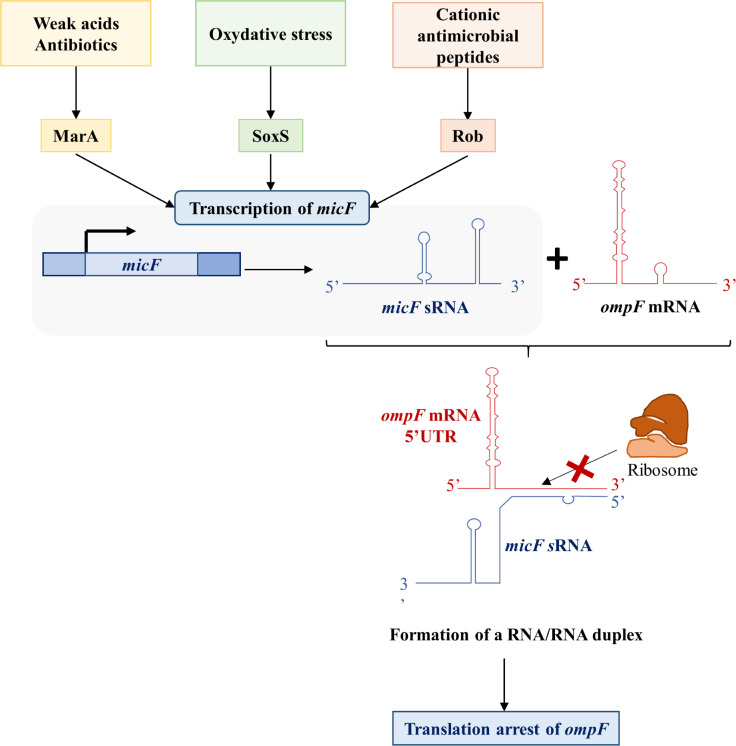
Post-transcriptional regulation of *ompF* expression in *Escherichia coli.* Post-transcriptional regulation of *ompF* by the formation of an RNA/RNA hybrid between the small regulatory RNA *micF* and *ompF* mRNA preventing the binding of the ribosome. Three positive regulators MarA, SoxS, and Rob, themselves activated by different environmental stimuli, activate transcription of *micF*.

Porin expression in *Enterobacteriaceae* can also be controlled at the post-transcriptional level (Fig 5). In *E. coli*, for example, expression of *ompF* is post-transcriptionally regulated through the action of the small regulatory RNA *mic*F, which binds to partially complementary sequences in the the 5′ untranslated region of *ompF* mRNA. This leads to the formation of an RNA/RNA hybrid that stops *ompF* translation by preventing the ribosome from binding. MicF is a significant regulator of OmpF [41,42]. Its expression is notably regulated by three positive transcriptional activators MarA, SoxS, and Rob [42,43]. These factors provide response to a wide variety of environmental stresses such as antimicrobial [42,44,45].

### Normal structure and regulation of porins involved in carbapenem entry in *P. aeruginosa*

Many porins have been identified in *P. aeruginosa* [46]. However, *P. aeruginosa* is characterized by the very low permeability of its outer membrane (about 8% of that of *E. coli*), which contributes (at least in part) to this bacterium’s high intrinsic resistance to antibiotics [46,47]. The reasons for this low permeability are partly explained by the absence of large general porins, such as *E. coli* OmpF, and OmpC and the presence of numerous substrate-specific porins.

Porins involved in carbapenems entry in *P. aeruginosa* are specific-porins. The specific OprD porin has attracted much interest in clinical practice as it is the main route by which carbapenems enter *P. aeruginosa* [48]. Initially, the role of this porin in the diffusion of carbapenems through the outer membrane of *P. aeruginosa* was suspected when it was observed that some clinical imipenem-resistant isolates of *P. aeruginosa* lacked a 45–46 kDa OMP compared to susceptible isolates [49,50]. This molecular weight range was characteristic of proteins of the D group according to the nomenclature of *P. aeruginosa* OMPs [51]. Further experiments showed that in fact two major OMP belonged to the D group: protein D1 and protein D2 [52]. The protein D2 (and not D1) was then identified as the porin allowing the diffusion of carbapenems through the outer membrane of *P. aeruginosa* and found to be associated with carbapenem-resistance when absent [48]. In 1990, Hancock and colleagues proposed the use of the name OprD, for outer membrane protein D, to designate the protein D2 and OprB to designate the protein D1 [53]. In 2012, OprD was then renamed as OccD1 for outer membrane carboxylate channels D1, but the name OprD remains still commonly used [54]. This porin is part of a 19-member family of OMPs in *P. aeruginosa*, the Occ family, distributed into two phylogenetic subfamilies: OccD is involved in the uptake of positively charged residues, and OccK, involved in the uptake of negatively charged cyclic molecules [46,54–57]. OprD is the archetypal member of the Occ family. It enables the transport of small nutrients that are essential for the bacteria, such as basic amino acids (arginine, lysine, histidine, and ornithine) and small basic peptides that contains these amino acids [48,58].

**Fig 6:**
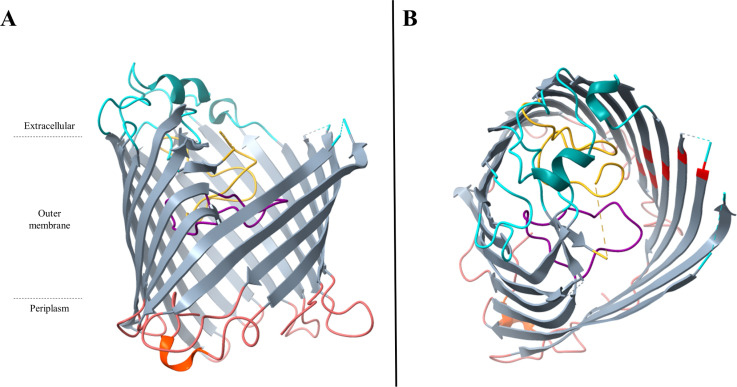
Overall structure of the OprD porin of *Pseudomonas aeruginosa.* **A**. Side view of OprD monomer unit. **B**. View of the OprD monomer unit from the top. Gray, β-strands; turquoise, extracellular loops and extracellular α-helices; orange, short periplasmic turns and periplasmic α-helices; magenta, pore-constricting loop L3; gold, pore-constricting loop L7 with dotted lines representing the L7 segment not visible on the structure; red, residues composing the basic ladder: Lys 375, Arg 391, Arg 389, Arg 30, and Arg 39 (from left to right). These graphics are based on PDB file 3SY7 and were drawn by using the program UCSF ChimeraX 1.8 (https://www.rbvi.ucsf.edu/chimerax) [22].

With regard to the protein structure, OprD monomer unit comprises 18-stranded β-barrel, nine loops, and a central channel constricted by two long extracellular loops (L3 and L7) (Fig 6A and 6B) [59]. The external loops 2 and 3 were determined to be entrances for basic amino acids and binding sites for imipenem [60,61]. OprD is still commonly considered a monomer. However, its X-ray crystal structure revealed the presence of two short β-strands characteristic of trimeric outer membrane channels, suggesting that OprD monomer units may actually form labile trimers within the outer membrane of *P. aeruginosa* [59]. Biswas and colleagues reinforced this notion by using non-denaturing polyacrylamide gel electrophoresis, which showed that OprD most likely forms trimers [59]. Although another crystal study with higher resolution confirmed Biswas and colleagues’ description, further studies are required to definitively elucidate the organization of OprD monomer units within the outer membrane of *P. aeruginosa* [54].

**Fig 7:**
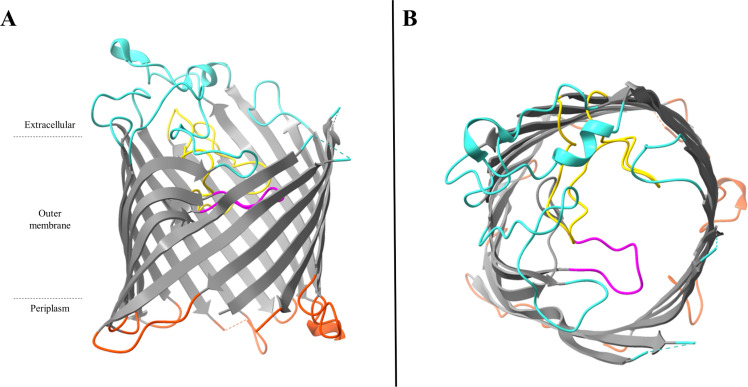
Overall structure of the OpdP porin of *Pseudomonas aeruginosa.* **A**. Side view of OpdP monomer unit. **B**. View of the OpdP monomer unit from the top. Gray, β-strands; turquoise, extracellular loops and extracellular α-helices; orange, short periplasmic turns and periplasmic α-helices; magenta, pore-constricting loop L3; gold, pore-constricting loop L7; dotted lines representing segment not visible on the structure. These graphics are based on PDB file 3SYB and were drawn by using the program UCSF ChimeraX 1.8 (https://www.rbvi.ucsf.edu/chimerax) [22].

Interestingly, OpdP (OccD3) shows the highest percentage of homology with OprD (OccD1) (51%), which suggests that one can compensate for the other in the uptake of nutrients (Fig 7). This hypothesis was confirmed by the observation that in the presence of arginine as a carbon source, wild type (WT) and single mutants have approximatively the same growth, whereas the double Δ*oprDΔopdP* mutant grew less well [62].

Other specific features of OprD are involved in the channel’s selectivity: a positively charged basic ladder (residues on the barrel wall), and an electronegative pocket due to predominantly negative charges in the pore-constricting loops (Fig 6B). These create an electrostatic pathway that probably guides the substrates through the pore [59]. Interestingly, OccD family members have also dynamic structures; for example, conformational changes in OccD proteins (OccD1–3) are required for the passage of substrates [54]. Molecular dynamics simulations of OccD1 have highlighted the conformational flexibility of the channel’s eyelet and the frequent formation of a relatively wide channel that probably allows carbapenem antibiotics to pass through [57].

**Fig 8:**
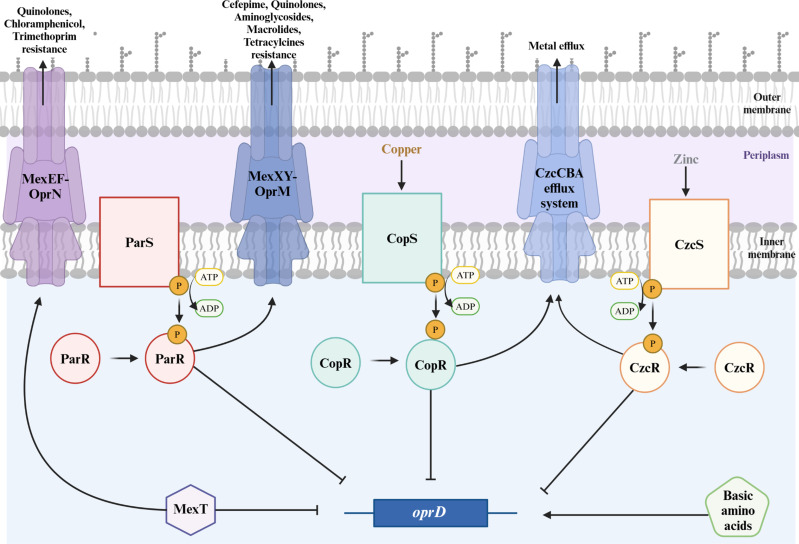
Regulatory network of *oprD* expression in *Pseudomonas aeruginosa.* The expression of *oprD* is positively regulated by basic amino acids such as arginine [72] and negatively regulated by the transcriptional regulator MexT and three two-component systems: ParRS, CopRS (induced by Copper) and CzcRS (induced by Zinc). In addition, MexT, activates the expression of MexEF-OprN, ParRS activates the expression MexXY-OprM, both leading to antimicrobial resistance and CopRS and CzcRS activate the expression of the CzcCBA efflux system, leading to metal resistance. Created with BioRender.com.

The regulation of OprD porin expression relies on a complex network involving two TCSs CzcRS and CopRS which downregulate OprD porin expression (Fig 8). Trace metals such as zinc (CzcRS) and copper ions (CopRS) induce them. Interestingly, CzcRS and CopRS jointly regulate both OprD expression and the entry of zinc and copper into the bacteria by increasing the expression of a metal efflux pump (CzcCBA efflux system), which leads to metal resistance [63–65]. A third TCS, ParRS, was described to downregulate OprD porin expression and upregulate the efflux system MexXY-OprM [66]. In addition, MexT, a positive regulator of the multidrug efflux system MexEF-OprN is also involved in the downregulation of OprD [67–69]. *P. aeruginosa* has the ability to form strong biofilms during infections [70]. Interestingly, in biofilms produced by *P. aeruginosa*, a strong decrease of *oprD* expression has been reported by Tata and colleagues [71]. This decrease of *oprD* expression might limit the efficiency of carbapenems in infections caused by *P. aeruginosa*.

### Mechanisms leading to porin loss or alteration in *Enterobacteriaceae* and *P. aeruginosa*

**Fig 9:**
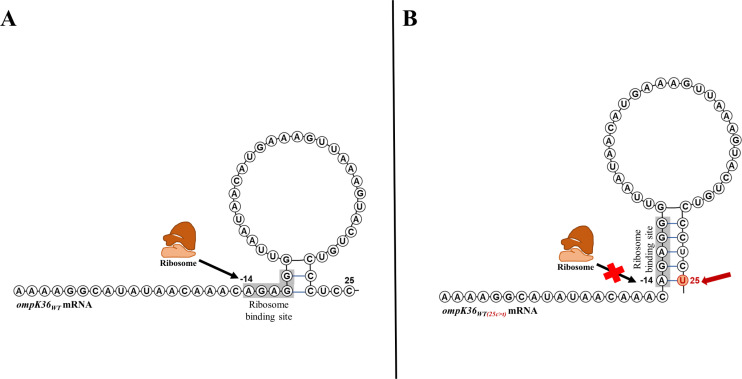
A schematic representation of the change in the secondary structure of *ompK36* mRNA caused by a 25c > t transition. **A**. The usual conformation of o*mpK36*_*WT*_ mRNA. **B**. The 25c > t transition leads to an intra-mRNA interaction between the uracil at position 25 (the red arrow) and the first adenine of the ribosome binding site at position −14. This specific interaction induces the formation of a stem structure that prevents the binding of the ribosome to the ribosome binding site (highlighted in gray), and thus restricts translation initiation. The figure was adapted from Wong *and colleagues* [75].

A wide range of “quantitative” changes (changes in the porin expression) or “qualitative” changes (changes in the porin structure) can lead to impermeability. In *Enterobacteriaceae*, several mechanisms leading to “quantitative” changes of general porins involved in carbapenem entry have been reported. They can be due to mutations in the coding sequence of the porin or in its promoter region or in the Shine-Dalgarno sequence (SDS), also called the ribosome-binding site. Indeed, these changes can lead to a frameshift or a premature termination codon affecting the transcription [73,74], or can prevent the transcription or translation initiation [75]. By way of an example of the latter type of mutation, the 25c > t transition in the SDS of the *ompK36* gene (Fig 9). Wong and colleagues found this transition in carbapenem-resistant clinical *K. pneumoniae* isolates. They showed that this leads to the formation of a stem structure in the porin mRNA that blocks the ribosome’s subsequent binding to the SDS, and disrupts translation [75]. Similarly, in *P. aeruginosa*, decrease of OprD porin expression is a frequent determinant of carbapenem resistance in non-metallo-β-lactamase carbapenem-resistant *P. aeruginosa* [76–80]. As for *Enterobacteriaceae*, OprD loss or decrease can result from mutations in the coding sequence of the porin gene leading to a frameshift or a premature stop codon or results from mutations in the upstream region of the *oprD* coding region [81–84].

Mutations in genes encoding factors that regulate porin expression can also decrease porins expression. For example, Dupont and colleagues identified that the resistance to ertapenem of an ESBL-producing *E. coli* clinical isolate resulted from a 188g>t substitution in the *ompR* gene, an important regulator gene for general porin expression in *Enterobacteriaceae* [85]. Similarly, mutations in the *cpxA* gene, encoding the inner membrane sensor kinase of the CpxRA TCS, have also been reported in carbapenem-resistant clinical enterobacterial isolates [86,87] or associated to an increase of carbapenem minimal inhibitory concentrations (MICs) in a *Serratia marcescens* clinical isolate [88]. Similar mutations have been described in pathways involved in the regulation of OprD expression. Indeed, mutations in *czcS*, a gene encoding the sensor protein of the CzcRS TCS, have been shown to decrease *oprD* expression [64]. Decreased *oprD* expression has also been reported in isolates of *P. aeruginosa* with mutational alterations of the ParRS TCS or with mutations in *mexS*, a gene immediately upstream of *mexT*, which acts as a suppressor of MexT [66,89,90].

Lastly, certain “qualitative” mutations alter the conformation of porins and therefore reduce their permeability. In *Enterobacteriaceae*, mutations of residues lining the channel or located in the constriction region and particularly in the loop L3 of general porins (e.g., OmpF and OmpC), were shown to reduce antibiotic permeability probably due to different steric hindrance or changes in charge distribution through the porin [91–93]. These structural changes can contribute to carbapenem resistance. A well-characterized structural change is the Gly115-Asp116 (GD) insertion in L3 of OmpK36 in *K. pneumoniae* ST258 [94]. Interestingly, the crystallographic structure of this porin variant shows that the GD insertion extends L3, which reduces importantly the pore (the pore diameter is reduced by 26%), restricting the diffusion of meropenem [94]. Concerning *P. aeruginosa*, structural mutations in external loop 2 of OprD can have a major impact on carbapenem permeability and lead to carbapenem resistance because this loop act as binding site for imipenem [56,60,61,95,96]. Thus, transformation of an OprD-deficient background *P. aeruginosa* with a plasmid encoding an OprD protein with deletion in loop L2 resulted in an 8-fold increase in the carbapenem MICs, compared to the parental strain transformed with a plasmid encoding the WT gene [96]. In addition, similar experiments showed that deletion in loop L3, also involved in imipenem passage through OprD, provided imipenem resistance [60].

## Impact of porin deficiency on carbapenem susceptibility

### Impact of porin deficiency on carbapenem susceptibility in *Enterobacteriaceae*

The relationship between impermeability and carbapenem resistance is complex and still poorly understood. Surprisingly, although general porins are major porins in *Enterobacteriaceae* in allowing access of carbapenems to their periplasmic targets, loss, or alteration of one of these porins, without additional resistance mechanism, has no effect on carbapenem susceptibility (Table 1). This has been demonstrated in different phenotypic studies, which assessed the individual contribution of porin loss on carbapenem resistance in laboratory strains. For example, using isogenic *K. pneumoniae* strains (derived from *K. pneumoniae* NVT2001), Tsai and colleagues demonstrated that deletion of only one general porin (OmpK35 or OmpK36) did not influence significantly susceptibility to imipenem (MIC = 0.25 mg/L in the WT, the Δ*ompK35* and the Δ*ompK36* strains) and meropenem (MIC = 0.003 mg/L in the WT strain and MIC = 0.006 mg/L in the Δ*ompK35* and the Δ*ompK36* strains) [97]. Similar results have been obtained in laboratory strains of *E. coli* (*E. coli* JF701 and JF703) [16]. Although a single deletion has no effect on susceptibility to carbapenems, changes for ertapenem particularly, have been noticed in ‘double’ general porin mutants. In *E. coli* HB4, the simultaneous deletion of both OmpF/OmpC resulted in resistance to ertapenem (MIC of ertapenem = 1 mg/L in the ‘double’ mutant versus 0.006 and 0.004 in the single OmpF (*E. coli* JF703) and OmpC (*E. coli* JF701) mutants, respectively), while MICs of imipenem and meropenem remained unchanged [16]. In *K. pneumoniae* (ATCC 13883 strain), an increase of the MIC of ertapenem has also been reported in the Δ*ompK35*Δ*ompK36* strain compared to the WT, Δ*ompK35* and Δ*ompK36* strains [37]. In addition, in *K. pneumoniae* (ATCC 13883 and NVT2001 strains), susceptibility to meropenem seems also to be moderately affected by the simultaneous lack of OmpK35/OmpK36 [37,97]. However, this simultaneous deletion was not enough to confer resistance to meropenem, according to the European Committee on Antimicrobial Susceptibility Testing’s breakpoints 2024. This weak impact of porin deficiency on carbapenem susceptibility can be explained by the presence of alternative porins that could compensate the function of lost major porins [98–100]. For example, in *K. pneumoniae* (CSUB10 strains), overexpression of the maltodextrin-specific porin, LamB, is essential to compensate the lack of both OmpK35/OmpK36 [99]. Interestingly, the loss of LamB in the OmpK35/OmpK36 mutant was associated with the expression of a new protein, probably another porin that could compensate the simultaneous absence of LamB/OmpK35/OmpK36 [99]. These results highlight the existence of a probable complex and evolving secondary network of porins, which could take over from the major porins when they are lost.

**Table 1:** Impact of porin deficiency and β-lactamase production on carbapenem MICs for laboratory strains of *Klebsiella pneumoniae* and *E. coli.*

Bacterial strains	Antibiotics, MICs (mg/L)			References
	Ertapenem	Imipenem	Meropenem	
***Escherichia coli***				
*E. coli* WT^a^	0.006	0.125	0.032	[14]
*E. coli* Δ*ompF*^b^	0.006	0.125	0.032	[16]
*E. coli* Δ*ompC*^c^	0.004	0.125	0.032	
*E. coli* Δ*ompC* + Δ*ompF*^d^	**1**	0.25	0.032	
*E. coli* Δ*ompC* + Δ*ompF* + CMY-2^d^	**256**	**32**	*8*	[14]
***K. pneumoniae***				
***K. pneumoniae* WT ± β-lactamase**				
*K. pneumoniae* WT^e^	0.015	0.25	0.03	[37,97]
*K. pneumoniae* WT + CTX-M-15^e^	0.25	1	0.125	[37]
*K. pneumoniae* WT + CMY-2^f^	ND	0.5	0.06	[97]
*K. pneumoniae* WT + CTX-M-14^f^	ND	0.25	0.06	[97]
*K. pneumoniae* WT + KPC-2^e^	**16**	**8**	*8*	[37]
***K. pneumoniae* Δ*ompK35* ± β-lactamase**				
*K. pneumoniae* Δ*ompK35*^e,f^	0.03	0.25	0.03–0.06	[37,97]
*K. pneumoniae* Δ*ompK35* + CTX-M-15^e^	0.5	1	0.125	[37]
*K. pneumoniae* Δ*ompK35* + CMY-2^f^	ND	0.5	0.06	[97]
*K. pneumoniae* Δ*ompK35* + CTX-M-14^f^	ND	0.25	0.06	[97]
*K. pneumoniae* Δ*ompK35* + KPC-2^e^	**32**	**16**	**32**	[37]
***K. pneumoniae* Δ*ompK36* ± β-lactamase**				
*K. pneumoniae* Δ*ompK36*^e,f^	0.0625	0.25	0.06	[37,97]
*K. pneumoniae* Δ*ompK36* + CTX-M-15^e^	**1**	1	0.25	[37]
*K. pneumoniae* Δ*ompK36* + CMY-2^f^	ND	2	0.5	[97]
*K. pneumoniae* Δ*ompK36* + CTX-M-14^f^	ND	0.5	0.125	[97]
*K. pneumoniae* Δ*ompK36* + KPC-2^e^	**32**	**32**	**32**	[37]
***K. pneumoniae* Δ*ompK35*Δ*ompK36* ± β-lactamase**				
*K. pneumoniae* Δ*ompK36 +* Δ*ompK35*^e^^,^^f^	**1**	0.5	0.125–0.25	[37,97]
*K. pneumoniae* Δ*ompK36 +* Δ*ompK35* + CTX-M-15^e^	**8**	1	2	[37]
*K. pneumoniae* Δ*ompK36 +* Δ*ompK35* + CMY-2^f^	ND	*4*	2	[97]
*K. pneumoniae* Δ*ompK36 +* Δ*ompK35* + CTX-M-14^f^	ND	1	1	[97]
*K. pneumoniae* OmpK36GD *+* Δ*ompK35* + KPC-2^e^	**128**	**64**	**128**	[37]
***K. pneumoniae* OmpK36GD ± β-lactamase**				
*K. pneumoniae* OmpK36GD^e^	0.03–0.06	ND	0.03–0.06	[37]
*K. pneumoniae* OmpK36GD *+* CTX-M-15^e^	**1**	1	0.25	
*K. pneumoniae* OmpK36GD *+* Δ*ompK35* + CTX-M-15^e^	**4**	1	1	
*K. pneumoniae* OmpK36GD + KPC-2^e^	**32**	**16**	**16**	
*K. pneumoniae* OmpK36GD *+* Δ*ompK35* + KPC-2^e^	**128**	**64**	**128**	

OmpK36GD corresponds to a structural change of the porin OmpK36, resulting from an insertion of two amino acids (a glycine at position 115 and an aspartic acid at position 116) in the loop L3, the porin still being produced. Δ*ompK36*, Δ*ompK35*, Δ*ompC*, Δ*ompF* corresponds to the deletion of *ompK36*, *ompK35*, *ompC*, and *ompF*, respectively. Boldface numbers indicate “resistant” strains and numbers in italics and underline indicate “susceptible, increased exposure” strains according to the European Committee on Antimicrobial Susceptibility Testing’s breakpoints 2024 (i.e., Ertapenem: S ≤ 0.5, R > 0.5; Imipenem: S ≤ 2, R > 4; Meropenem: S ≤ 2, R > 8).

Bacterial strains:

^a^*E. coli* TOP10;

^b^*E. coli* JF703;

^c^*E. coli* JF701;

^d^*E. coli* HB4;

^e^*K. pneumoniae* ATCC 13883.

^f^*K. pneumoniae* NVT2001.

ND, no data. MICs, minimal inhibitory concentrations. WT, wild type.

In practice, loss or alteration of porins has more impact on carbapenem susceptibility when the absence of porin is associated with additional resistance mechanisms such as the production of β-lactamase (Table 1). Impermeability and β-lactamase production amplify each other. Thus, loss or alteration of porins leads to an increase of the carbapenems MICs in β-lactamase-producing or overproducing isolates. This is particularly true when the two major porins are simultaneous deleted [97]. For example, in an isogenic *K. pneumoniae* strain (NVT2001), MICs of imipenem were ≤0.5 mg/L in the non-porin-deleted strains with or without CTX-M-14 or CMY-2 β-lactamases production, and were of to 1 mg/L in the Δ*ompK35ΔompK36* strain expressing CTX-M-14 and up to 4 mg/L in Δ*ompK35ΔompK36* strain expressing CMY-2 [97]. Not surprisingly, these results also show that the carbapenems MICs are differently affected according to the hydrolysis spectrum of β-lactamases associated with the porin deficiency [14,16,97]. Whatever in *K. pneumoniae* or *E. coli*, MICs of carbapenems were higher when the deletion of porins was combined with CMY-2 than other tested β-lactamases such as CTX-M-14 (in *K. pneumoniae* NVT2001) or ACT-1, DHA-1, ACC-1, and FOX-1 (in *E. coli* HB4) [14,97]. Similarly, in a dual-porin-deficient *E. coli* (HB4), expression of an extended spectrum cephalosporinase conferred a higher level of resistance to ertapenem than a narrow-spectrum cephalosporinase (32 mg/L versus 2 mg/L, respectively) [16]. Porin deficiency also accentuated the carbapenem resistance conferred by carbapenemases [37]. In a KPC-producing *K. pneumoniae* (ATCC 13883 strain), the simultaneous lack of porins OmpK35 and OmpK36 led to a 16-fold increase in the MICs for meropenem and imipenem (128 mg/L when porins deletion was associated with KPC-production versus 8 mg/L in a KPC-producing isolate with no porin deletion) [37].

The association of both mechanisms: impermeability and β-lactamase production, is a frequent cause of carbapenem-resistance in *Enterobacteriaceae* clinical isolates, probably facilitated by the use of broad-spectrum antibiotic therapies [15]. In a multicentric French study, association of porin deficiency and β-lactamase was the main mechanism conferring carbapenem-resistance, much more frequent than carbapenemase production [15]. In general, all *Enterobacteriaceae* species are concerned by this and adapt with regard to their carbapenem permeability because they have similar general OmpC-like and OmpF-like porins. Indeed, many clinical reports described the emergence of carbapenem-resistant *Enterobacteriaceae* due to the combination of such resistance mechanisms in different species such as *E. coli* [101], *Citrobacter freundii* [102], *K. pneumoniae* [73], *K. aerogenes* [103–106], *Hafnia alvei* [107], *S. marcescens* [108] and *Enterobacter cloacae* [73]. However, to the best of our knowledge, reduced susceptibility to ertapenem and/or meropenem because of porin deficiency has not been reported in the *Morganellaceae*, but members of this family have a naturally low susceptibility to imipenem because of the presence of low-affinity PBPs for imipenem, the PBP2 [109].

Interestingly, porin structural alterations can also have a slight impact on the carbapenem susceptibility. For example, *K. pneumoniae* strains (ATCC 13883) harboring an OmpK36 variant with a GD structural insertion in the third loop, had up to a 4-fold increase in ertapenem MIC compared to the parental strain (MIC = 0.06 mg/L versus 0.015 mg/L, in the OmpK36GD and WT strains, respectively) [37]. The effect of OmpK36GD was quite similar to that induced by the lack of OmpK36 with regard to the MICs for carbapenems even when associated with expression of different β-lactamases [37].

Lastly, in clinical or laboratory *Enterobacteriaceae* strains, susceptibility to ertapenem seems more affected by porin deficiency than imipenem and meropenem, whether isolated or associated with β-lactamase production. This may be related to its larger size and its more negative charges that probably slow down its penetration through porins [13,110,111].

### Impact of porin deficiency on carbapenem susceptibility in *P. aeruginosa*

The naturally reduced permeability of the outer membrane of *P. aeruginosa* and the presence of active efflux pumps systems are partly responsible for the poor in vitro activity of ertapenem against this bacterium [112,113]. Therefore, this carbapenem is not considered clinically useful for the treatment of infections caused by *P. aeruginosa*. In contrast, imipenem and meropenem are more active [112,114]. The relationship between loss or alteration of OprD and imipenem and/or meropenem-resistance has been the subject of numerous publications, as it is frequent in clinical carbapenem-resistant isolates of *P. aeruginosa* [77,78,80,115–117].

Nevertheless, the real impact of OprD deletion on carbapenem susceptibility is difficult to assess as *P. aeruginosa* naturally expresses many mechanisms which can play a role in reducing the susceptibility to carbapenems (e.g., inducible chromosomal AmpC β-lactamas—also called *Pseudomonas*-derived cephalosporinase—and active efflux pumps systems). For example, Mushtaq and colleagues studied two different strains (*P. aeruginosa* 1405 and 2297) [114]. For one (*P. aeruginosa* 1405), when only tiny amounts of the chromosomal AmpC β-lactamase are produced, loss of OprD did not result in increased MICs of meropenem and imipenem (Table 2) [114]. Conversely, for the second strain (*P. aeruginosa* 2297), in similar AmpC-basal expression, loss of OprD resulted in a 32-fold increase in meropenem MICs (0.125 mg/L versus 4 mg/L, in WT and *ΔoprD*, respectively) and in a 8-fold increase in imipenem MICs (0.25 mg/L vsersu 2 mg/L, in *oprD*_*WT*_ and *ΔoprD*, respectively). However, these changes were insufficient to increase MICs above the resistant breakpoint according to the European Committee on Antimicrobial Susceptibility Testing’s breakpoints 2024 [114]. Additional mechanisms are necessary to contribute to carbapenem resistance in OprD-deficient *P. aeruginosa*. Indeed, Mushtaq and colleagues demonstrated that when the chromosomal AmpC β-lactamase was derepressed (i.e., expressed copiously without induction) in OprD-deficient mutants of *P. aeruginosa* 1405 and 2297, changes in carbapenem MICs pushed them above the resistant breakpoint for imipenem, and led for resistance or reduced susceptibility for meropenem (Table 2) [114]. Conversely, derepression of AmpC β-lactamase alone did not change susceptibility to imipenem and meropenem (Table 2). Livermore previously found similar data (Table 2) [118]. These results highlight the importance of the interplay between AmpC β-lactamase and impermeability in *P. aeruginosa* strains to cause carbapenem resistance. Indeed, in OprD-deficient strains; low amounts of carbapenems reaching the cytoplasm are rapidly hydrolyzed by the inducible chromosome-borne AmpCβ-lactamase leading to carbapenem resistance. Interestingly, imipenem seems a little bit more affected by the AmpCβ-lactamase in porin deficient strains, than meropenem, probably because the meropenem is more stable than imipenem to AmpC β-lactamase hydrolysis [115,119].

**Table 2:** Impact of porin deficiency and β-lactamase production on carbapenem MICs for laboratory strains of *Pseudomonas aeruginosa.*

Bacterial strains	*oprD*	Antibiotics, MICs (mg/L)		References
		Imipenem	Meropenem	
*P. aeruginosa* (strain 1405)	WT	*0.25–0.5*	0.12–0.25	[114,118]
	Δ*oprD* and Increased AmpC expression	**16**	*4* **–16**	
*P. aeruginosa* (strain 2297)	WT	*0.12–0.25*	0.125–0.25	
	*ΔoprD* and Increased AmpC expression	**16**	2–*8*	

MICs, minimal inhibitory concentrations; WT, wild type; Δ*oprD* corresponds to mutants lacking OprD. Boldface numbers indicate “resistant” strains (for meropenem, interpretations are based on breakpoints for indications other than meningitis) and numbers in italics and underlines indicate “susceptible, increased exposure” strains according to the European Committee on Antimicrobial Susceptibility Testing’s breakpoints 2024 (i.e., Imipenem: 0.001 mg/L < Susceptible, increased exposure ≤ 4 mg/L, Resistant > 4 mg/L; Meropenem: S ≤ 2 mg/L, 2 mg/L < Susceptible, increased exposure ≤ 8 mg/L, Resistant > 8 mg/L).

More recently, OpdP (also called OccD3), a porin that shares a high degree of similarity with OprD, has attracted interest [62,120]. Using a transposon mutant library and then isogenic mutants’ constructions, Isabella *et al.* demonstrated that this porin could also be involved in carbapenems uptake [120]. However, the inactivation of OpdP alone did not result in a significant increase in carbapenem MICs. Nevertheless, what is interesting is the impact of the simultaneous deletion of both OprD and OpdP in a *P. aeruginosa* PAO1 reference strain. Indeed, in minimal media, it led to a significant increase of carbapenems MICs (16- to 64-fold compared to the parental WT strain for imipenem and meropenem, respectively). These raises reduced susceptibility to imipenem but were insufficient to cause meropenem resistance. In contrast, in rich media, deletion of both OprD and OpdP led to a less important increase of carbapenems MICs (8- to 16-fold compared to the parental WT strain, for meropenem and imipenem, respectively), but these changes were sufficient to provide resistance to imipenem (MIC = 16 mg/L for the ‘double’ mutant *vs* 1 mg/L for the WT strain) and to reduce susceptibility to meropenem (MIC = 4 mg/L for the ‘double’ mutant *vs* 0.5 mg/L for the WT strain) [120]. These data suggest that the simultaneous absence of OprD and OpdP could also contribute to carbapenem resistance. However, further studies are needed to definitively evidence the role of the loss of OpdP in carbapenem-resistance and to assess its frequency in clinical isolates.

Usually, carbapenem resistance in *P. aeruginosa* is driven by multifactorial mechanisms [78,121]. Many different mechanisms can be involved in addition to loss of OprD, such as hydrolyzing enzymes (e.g., metallo-β-lactamases), increased production of AmpC β-lactamase, active efflux pumps systems (e.g., MexAB-OprM, MexXY-OprM…). These mechanisms are often seen in conjunction in clinical isolates. The combination of loss of OprD and overproduction of AmpC β-lactamase is very frequent, particularly in emergent multidrug resistant and extensively drug resistant *P. aeruginosa* strains, also called ‘high-risk clones’ [78,121]. These two mechanisms were found together in 79% of XDR clinical *P. aeruginosa* strains of a large collection [121]. All these findings reveal the complexity of underlying mechanisms, which contribute to carbapenem resistance in *P. aeruginosa*. So that, impermeability must be considered as a factor within a dynamic interplay of many different mechanisms contributing to carbapenem resistance.

## Impact of porin deficiency on fitness and virulence

### Impact of porin deficiency on fitness and virulence in *Enterobacteriaceae*

Fitness is related to the natural lifestyle of a microorganism within a given context. It can be defined as the ability of a microorganism to adapt its metabolism to survive in different conditions (different physicochemical conditions, nutrient availability, presence of antibiotics…). Virulence is related to the interaction of a microorganism with its host. It can be defined as the ability of a microorganism to invade the host (*via* adhesion to epithelial cells), survive the host’s immune defenses, disseminate, and cause the host’s death.

Many researchers have attempted to determinate the bacterial physiological fitness cost due to loss of porin in *Enterobacteriaceae*. In *K. pneumoniae* (ATCC 13883, 10.85 and 11.76 strains), Fajardo-Lubián and colleagues showed that individual loss of the major porin OmpK35 or OmpK36 has no significant impact on the bacterial growth rate in rich media [37] (Table 3). Similar results have also been found by Tsai and colleagues and Chen and colleagues [97,122]. Although the loss of one major porin alone has no impact on the bacterial growth, loss of both major porins affects it. Indeed, in rich media, Δ*ompK35*Δ*ompK36* mutants grew more slowly than their parental strains [37,97].

**Table 3:** Overview of the interplay between carbapenems resistance and fitness/virulence in porin-deficient: examples of *Klebsiella pneumoniae* and *Pseudomonas aeruginosa* strains.

	Impact on carbapenem susceptibility	Impact on fitness in vitro	Impact on fitness in vivo and virulence	References
**In *K. pneumoniae***				
**Loss of OmpK35**	No significant change in carbapenem MICs compared to the WT strain.	No impact on exponential phase growth in rich media. The ability of Δ*ompK35* mutants to compete against the WT strain was only slightly affected in rich media, but strongly reduced in low-nutrients conditions.	No impact on the virulence. In in vivo competition experiments, Δ*ompK35* mutants were not disadvantaged compared to the WT strain. In a mouse model of peritonitis, Δ*ompK35* mutants were as virulent as the parental strain.	[37,97,122]
**Loss of OmpK36**	No significant change in imipenem and meropenem MICs compared to the WT strain. However, a slight increase in ertapenem MIC (4-fold) compared to the WT strain was observed, but OmpK36-deficient strains were still susceptible to ertapenem.	No impact on exponential phase growth in rich media. Δ*ompK36* mutants were rapidly outcompeted by the WT strain in rich media, but their ability to compete with the WT strain was only slightly affected in low-nutrient conditions.	Loss of OmpK36 confers a lower virulence in vivo. In in vivo competition experiments, Δ*ompK36* mutants were strongly disadvantaged compared to the WT strains. These mutants were also more susceptible to neutrophil phagocytosis and more rapidly cleared from the liver in a mouse model of peritonitis. Δ*ompK36* mutants were less lethal than the parental strain in this model.	[37,97,122]
**Loss of OmpK35 and OmpK36**	Per se, the isolated loss of OmpK35 and OmpK36 led to a resistance to ertapenem and a slight increase (8-fold) in meropenem MICs compared to the WT strain, but double mutants were still susceptible to meropenem. In combination with ESBL- or AmpC-production, loss of OmpK36 and OmpK35 led to a greater increase in imipenem and meropenem MICs (16-fold and 66-fold, respectively, in association with CMY-2, thus altering imipenem susceptibility).	Δ*ompK35ΔompK36* mutants have a significant growth defect compared to their isogenic parents and were not able to compete in vitro with them.	Loss of both porin OmpK35 and OmpK36 led to a lower virulence. In in vivo competition experiments, Δ*ompK35*Δ*ompK36* mutants were strongly disadvantaged compared to the WT strain. Δ*ompK35*Δ*ompK36* mutants were less virulent than their parental strain in vivo.	[37,94,97]
**OmpK36GD**	OmpK36GD phenotype is responsible for a slight increase in ertapenem MIC (4-fold) compared to the WT strain but OmpK36GD strains were still susceptible to ertapenem. No significant change in meropenem MICs compared to the WT strain. On the other hand, it magnifies the carbapenemase activity exhibited by ESBL and KPC-enzymes, thus leading to increased imipenem and meropenem MICs (4-fold and 8-fold, respectively, in association with CTX-M-15).	Better ability of OmpK36GD strains to compete against the WT strain compared to OmpK36-deficient strains.	In vivo competition experiments showed that the OmpK36GD phenotype confers an advantage compared to the OmpK36-deficient phenotype. In an in vivo model of ventilator-associated pneumonia, *ompK36GD* strains were disadvantaged compared to the WT, but were advantaged compared to the Δ*ompK36* mutants.	[37]
**In *P. aeruginosa***				
**Loss of OprD**	Variable effect on imipenem and meropenem MICs.	Controversial data ranging from “no effect” on fitness to “enhanced fitness”.	Controversial data ranging from “a slight attenuated virulence” or “no change in virulence” to an “enhanced virulence” (more resistant to killing by acidic pH or normal human serum, higher lethality in mice infected with OprD-mutants compared to mice infected with the WT strain in a mouse model of pneumonia).	[18,114,123–125]

MIC(s), minimal inhibitory concentration(s); ESBL, extended spectrum β-lactamase.

In vitro competition experiments between parental strains and porin-deficient mutants, in different media, also allowed to characterize the impact of loss of porin on bacterial fitness. The ability of mutants to compete with their isogenic parents differs according to the mutants and the conditions [37]. In rich media, Δ*ompK36* strains (from *K. pneumoniae* ATCC 13883, 11.76 and 10.85 strains) were rapidly outcompeted by the WT strains and accounted for only 20% of the total combined population on day 3. In contrast, the ability of Δ*ompK35* strains (derived from *K. pneumoniae* ATCC 13883, 11.76 and 10.85 strains) to compete with the WT strains was only slightly affected in these conditions. However, the opposite is true under low nutrient conditions. In these conditions, Δ*ompK35* mutants (*K. pneumoniae* ATCC 13883 mutants) were rapidly outcompeted by the WT strains contrary to Δ*ompK36* strains (*ΔompK36-*ATCC 13883) [37]. This emphasizes the importance of OmpK36 in high-osmolarity, high-nutrient media (such as in the gastro-intestinal tract, these enterobacteria natural environment in humans) and of OmpK35 in nutrient-limited conditions. Not surprisingly, Δ*ompK35*Δ*ompK36* mutants were not able to compete with their isogenic parents and accounted for almost 0% of the total population on day 3 in rich media (*ΔompK35ΔompK36-*ATCC 13883). Globally, these results suggest that loss of porin could render *Enterobacteriaceae* less fit depending on conditions in which they grow.

Loss of certain porins could also have an effect on bacterial virulence. In in vitro assays, Δ*ompK36* mutants (*ΔompK36-*NVT1002) were more susceptible to neutrophil phagocytosis than their parental strain (NVT1002) [122]. The reduced virulence of OmpK36-deficient strains was also confirmed in animal model studies. Indeed, Chen and colleagues demonstrated that *ΔompK36* mutants (*ΔompK36-*NVT1002) were more rapidly cleared from the liver after intraperitoneal injection and were 100 times less lethal than their parental strain (NVT1002) [122]. In addition, competition experiments in a mouse model of gastrointestinal tract colonization showed that OmpK36-deficient bacteria (Δ*ompK36-*ATCC 13883) were strongly disadvantaged compared to their parental strain (ATCC 13883) [37].

OmpK36 might have a crucial role in adhesion and invasion of the digestive epithelium. Interestingly, in *E. coli*, OmpC, which is an OmpK36-like porin, is involved in the interaction between adherent-invasive *E. coli* (e.g., LF82) with intestinal epithelial cells, under high-osmolarity conditions, similar to that encountered in the gastrointestinal tract [8]. Indeed, increased expression of OmpC in LF82 adherent-invasive *E. coli* appears to activate the σ^E^ regulatory pathway, which in turn, induces the expression of genes encoding flagella, type 1 pili and others factors involved in adhesion and invasion [8]. Similarly, in *Shigella flexneri* (derived from the WT serotype 5 strain M90T), Bernardini and colleagues demonstrated that OmpC contributes significantly to virulence during invasion [126]. Taken as a whole, these data suggest strongly that OmpK36 is a virulence factor probably indirectly involved in adhesion and invasion via the activation of the σ^E^ regulatory pathway. Therefore, this could explain why OmpK36-deficient strains have a reduced virulence. Clinical isolates of *K. pneumoniae* exhibiting loss of OmpK36 alone are extremely rare probably due to the clearly disadvantage conferred by deletion of the porin [37].

However, loss of porins in *Enterobacteriaceae* is not always associated with a decrease in virulence. For example, experiments in a mouse model of peritonitis showed that OmpK35-deficient mutants were as virulent as the parental strains (NVT2001S), which was in line with the conserved fitness of Δ*ompk35* strains in rich media in vitro [97]. In addition, in vivo competition experiments have not evidenced an obvious disadvantage of the loss of OmpK35 [37]. Thus, these data suggest that deletion of OmpK35 has no effect on virulence and could constitute an evolutionary advantage for bacteria: contribute to antibiotic resistance without loss of virulence. Consistently, a high percentage of clinical isolates (nearly a third of the strains) appear to have lost their ability to express OmpK35 [37]. There is another example suggesting that loss of porin is not always associated with reduced virulence. Indeed, in adherent-invasive *E. coli* LF82, Rolhion and colleagues showed that deletion of OmpF (which is an OmpK35-like) is correlated with an increased invasion of intestinal epithelial cells [8]. Interestingly, they also demonstrated that this increased invasion of OmpF-deficient strains is linked with an increased expression of OmpC (OmpK36-like porin), probably to compensate the absence of OmpF [8]. These results suggest that the impact of loss of porin on virulence could also depend on the function and role in virulence of other increased porins involved to compensate the function of the porin lost.

Some researchers have stated that the cost of loss of porins in *Enterobacteriaceae* may be also partially offset by the expression of alternative outer membrane porins [37,98–100,127]. For example, the loss of both major porins (OmpK35 and OmpK36) resulted in increased expression of *phoE* and *lamB*, two genes encoding alternative porins in *K. pneumoniae* (ATCC 13883 and 10.85 strains) [37]. OmpK26 could also compensate for the absence of OmpK35/36 in the clinical *K. pneumoniae* KpCR-1 strain [98]. This questions about the compensatory effect of the alternative porins on the bacterial fitness and virulence. However, for example, although the expression of the porin OmpK26 partially compensated for the absence of OmpK35/36 in carbapenem-resistant *K. pneumoniae* (KpCR-1 strain), it did not fully restore the microorganism’s fitness in vitro or in vivo [98]. Furthermore, Fajardo-Lubián and colleagues’ experiments with isogenic *K. pneumoniae* strains showed that the phosphoporin PhoE and the maltodextrin channel LamB (the most important porins in the compensatory response when OmpK35 is lacking) are not efficient substitutes for OmpK36-deficient or dual OmpK-36/OmpK35-deficient strains (derived from ATCC 13883 and 10.85 strains) [37]. However, data are conflicting in the literature regarding the role of alternative porins to restore fitness and virulence in major porin-deficient strains. The results of a study suggested that overproduction of alternative porins could rescue the fitness cost caused by the loss of the two general porins [128]. Indeed, Knopp *et al.* showed that dysregulation of the Pho and Chip regulatory pathways leads to overproduction of the PhoE and ChiP alternative porins in double OmpC and OmpF mutants (derived from *E. coli* MG1655). This overproduction fully or partially compensated for the growth defect seen in the OmpC and OmpF double mutants [128]. However, PhoE overexpression in these mutants also restored susceptibility to ertapenem and meropenem [128]. It seems unlikely that overproduction of alternative porins can simultaneously restore fitness and maintain carbapenem resistance.

The absence of effective restoration of virulence by alternative porins may explain why *Enterobacteriaceae* lacking both major porins simultaneously were found to have reduced virulence. In experiments with *Caenorhabditis elegans*, Pantel and colleagues showed that the lack of both OmpC and OmpF in an uropathogenic *E. coli* MECB5 strain, representative of the ST131 *H*30-Rx subclone, was associated with reduced virulence. Indeed, the loss of these two general porins created a physiological disadvantage (decreased motility and ability to form biofilm) and low virulence (evidenced by a 50% increase in the killing time) [129]. Pantel and colleagues stated that the time course of biofilm formation, which is also an important pathogenicity factor, was slower in porin-deficient strains and that this accounted for the strains’ slower colonization of *C. elegans* [129]. The study’s results were in line with those of Dorman and colleagues, who showed that a mutation in *ompR* (a gene involved in the control of the expression of OmpC and OmpF), in a *Salmonella* Typhimurium strain (SL1344), significantly attenuated virulence in a mouse model of systemic infection [130]. Similarly, dual OmpK35/OmpK36 deficient *K. pneumoniae* mutants (derived from NVT2001S and ATCC 43816) also exhibited significantly lower virulence in a murine model of peritonitis and in a severe pneumonia model [94,97]. The same pattern of virulence was observed for *K. aerogenes* because imipenem-resistant clinical isolates lacking both Omp36 and Omp35 were significant less fit in a *C. elegans* model [105]. These results indicate that the deletion of both major porins leads to a significant decrease in pathogenicity of *Enterobacteriaceae*.

Structural changes in a porin can also affect the channel’s permeability and therefore may impact bacterial fitness and virulence. For example, a well-known structural change is the Gly115-Asp116 (GD) insertion in L3 of OmpK36 in *K. pneumoniae*, which extents this loop and constricts the pore. A carbapenem-resistant *K. pneumoniae* clinical isolate (ST258) lacking the OmpK35 porin and producing a modified OmpK36 with a GD insertion was shown to cause a fitness disadvantage in an in vivo model of ventilator-associated pneumonia, compared to others strains tested (OmpK35_WT_ or OmpK35_ST258_ and OmpK36_WT_ or OmpK36_ST258_) [94]. Similarly, Fajardo-Lubián and colleagues showed that ΔOmpK35/OmpK36GD mutants (ATCC 13883) were a little disadvantaged in vivo or in vitro compared to the WT strains. However, strains harboring OmpK36GD have a clear in vitro and in vivo fitness advantage in competition experiments, compared to the OmpK36-deficient strain [37]. Thus, this porin structural alteration appears to be an interesting solution for the bacteria to trade-off between carbapenem-resistance and fitness/virulence. Indeed, this alteration contributes to carbapenem resistance at low cost to colonizing ability, competitiveness, or pathogenicity. This L3 loop variation in OmpK36 is probably the result of a convergent evolutionary process. This example highlights the role of porin in successful adaptation to human colonization/infection and antibiotic resistance in *Enterobacteriaceae* [37].

### Impact of porin deficiency on fitness and virulence in *P. aeruginosa*

OprD-deficiency was previously thought to have no obvious effect on fitness and virulence of *P. aeruginosa* (Table 3). For example, growth rates in rich media of a clinical strain of *P. aeruginosa* O12 producing a penicillinase alone was equivalent to that of its isogenic variant with the same initial resistance associated with OprD impermeability [123]. In addition, when immunocompetent mice were infected with these two strains to cause a pneumonia, there was no statistically difference in the survival rate: almost all mice died in the two groups [123]. Wheatley and colleagues also showed that emerging *oprD* mutant clinical isolates of *P. aeruginosa* close to ST17, recovered over a 3-week period from patients suffering from pneumonia grew just as well as their ancestral strain and did not appear to be associated with a fitness costs in vitro [124]. Another study obtained slightly different results by showing that the deletion of OprD was associated with an attenuated virulence compared to its parental strain (PAO1) in a murine pneumonia model (at day 2, there was 100% of lethality in the group infected with the WT strain *vs* almost 50% of lethality in the group infected with the strain lacking OprD) [125]. However, emergence of methods combining large-scale transposon mutagenesis with high-throughput DNA sequencing strongly called into question these previous results, providing new insights for a comprehensive analysis of bacterial fitness and virulence.

Modern transposon-sequencing methods became important tools for assessing the fitness contribution of each gene of a bacterial genome within specific conditions [131]. Skurnik and colleagues used one of these methods (insertion-sequencing) to analyze the fitness of 300,000 mutants of *P. aeruginosa* PA14 in a mouse model for gut colonization and systemic dissemination [132]. They showed that transposon insertions, disrupting the *oprD* gene, led not only to carbapenem resistance, but also to an enhanced in vivo fitness compared to mutants lacking OprD transposon insertions [132]. Indeed, OprD porin-inactivating mutations have been found to drastically increase the mucosal colonization and dissemination to the spleen. Remarkably, this increase in fitness was only observed for insertions in *oprD.* In contrast, transposon insertions in all the other OMPs genes resulted in a decrease (rather than an increase) in in vivo fitness [132]. They also demonstrated that OprD-deficient strains were more resistant to killing by acidic pH or normal human serum and had increased cytotoxicity against murine macrophages. Important changes in the transcription of genes may contribute to this increase in virulence of OprD-mutants [132]. Consistent with these observations, Roux and colleagues found a higher lethality rate in mice infected with OprD-mutants compared to mice infected with the WT strain (PA14) in a mouse model of pneumonia. At day 2, they observed 100% of lethality in the group infected with the *oprD* transposon insertion mutants *vs* almost 40% of lethality in the group infected with the WT strain [18]. These findings indicate that inactivation of *oprD* could be responsible for a specific increased virulence of *P. aeruginosa*. Consequently, carbapenem therapy could select *P. aeruginosa oprD* strains resistant to carbapenem and with a potentially enhanced fitness and virulence. Interestingly, a report suggests that *P. aeruginosa oprD* mutants could also emerge without carbapenem treatment [133]. This is consistent with the advantageous fitness conferred by this phenotype, which could play a role in the selection of strains resistant to carbapenems.

## Conclusion

Porins are key bacterial OMPs whose total amount is tightly regulated. They are at the heart of a complex regulatory network, which aims to deliver vital nutrients to the bacteria cell within different conditions, trying to avoid the entry of toxic compounds like carbapenem antibiotics when the bacteria are exposed. Loss or alteration of porins contributes to carbapenem resistance, particularly when associated with other resistance mechanisms such as production of β-lactamases. These changes in porins are particularly of concern because they are part of the arsenal of clinical multidrug resistant *Enterobacteriaceae* and *P. aeruginosa* isolates. However, in return, they also affect bacterial fitness and virulence. Although many studies have shown that porin-deficiency frequently results in reduced fitness and virulence, other studies have instead revealed that loss or alteration of porin could increase bacterial virulence, such as OprD-inactivating mutations in *P. aeruginosa*. These discordant outcomes are probably due in part to the different methods used to explore bacterial fitness and virulence [134]. Many parameters can influence the results (strains used, their nutrient requirements, their natural lifestyle, their pathogenicity mechanisms, the animal model used…) and consequently, studies can fail to detect and quantify the fitness cost of carbapenem-resistant porin deficient strains. Recently, transposon-sequencing methods have emerged as methods of choice to study the role of gene inactivating mutations in bacterial fitness and virulence. While it has been reported that secondary mutations could explain the lack of fitness cost of some antibiotic resistance acquisitions [135], to our knowledge no secondary mutations were found in either *P. aeruginosa* or enterobacteria carbapenem resistant strains that would explain their preserved or even increased fitness. Further studies and further experimental procedures are still needed to provide a complete picture of the complex relationship between porin deficiency, carbapenem resistance and bacterial fitness and virulence. Finally, these data also highlight the effects of the selective pressure induced by carbapenems on the dynamics of the bacterial populations as we illustrated. The consequent emergence of multidrug resistant strains, reminding us how it is important to use these antibiotic treatments appropriately.
